# NT-proBNP or Self-Reported Functional Capacity in Estimating Risk of Cardiovascular Events After Noncardiac Surgery

**DOI:** 10.1001/jamanetworkopen.2023.42527

**Published:** 2023-11-08

**Authors:** Giovanna Lurati Buse, Jan Larmann, Hans-Jörg Gillmann, Katarzyna Kotfis, Michael T. Ganter, Daniel Bolliger, Miodrag Filipovic, Luca Guzzetti, Frédérique Chammartin, Eckhard Mauermann, Daniela Ionescu, Wojciech Szczeklik, Stefan De Hert, Beatrice Beck-Schimmer, Simon J. Howell

**Affiliations:** 1 Anesthesiology Department University Hospital Düsseldorf, Heinrich Heine University, Düsseldorf, Germany; 2 Department of Anesthesiology, Heidelberg University Hospital, Heidelberg, Germany; 3 Department of Anaesthesiology and Intensive Care Medicine, Hannover Medical School, Hannover, Germany; 4 Department of Anesthesiology, Intensive Therapy and Acute Intoxications, Pomeranian Medical University, Szczecin, Poland; 5 Department of Anesthesiology, Kantonsspital Winterthur, Winterthur, Switzerland; 6 Clinic for Anaesthesia, Intermediate Care, Prehospital Emergency Medicine and Pain Therapy, University Hospital Basel, Basel, Switzerland; 7 Division of Anesthesiology, Intensive Care, Rescue and Pain Medicine, Kantonsspital St Gallen, St Gallen, Switzerland; 8 Anesthesia and Intensive Care Department, University Hospital, Varese, Italy; 9 Division of Clinical Epidemiology, Department of Clinical Research, University Hospital Basel, University of Basel, Basel, Switzerland; 10 Department of Anesthesiology, Zurich City Hospital, Zurich, Switzerland; 11 Department of Anaesthesia and Intensive Care I, Iuliu Hatieganu University of Medicine and Pharmacy, Cluj-Napoca, Romania; 12 Center for Intensive Care and Perioperative Medicine Jagiellonian University Medical College, Kraków, Poland; 13 Department of Anaesthesiology and Peri-operative Medicine, Ghent University Hospital, Ghent University, Ghent, Belgium; 14 Institute of Anaesthesiology, University Hospital Zurich, University of Zurich, Zurich, Switzerland; 15 Leeds Institute of Medical Research at St James’s, University of Leeds, Leeds, United Kingdom

## Abstract

**Importance:**

Nearly 16 million surgical procedures are conducted in North America yearly, and postoperative cardiovascular events are frequent. Guidelines suggest functional capacity or B-type natriuretic peptides (BNP) to guide perioperative management. Data comparing the performance of these approaches are scarce.

**Objective:**

To compare the addition of either N-terminal pro-BNP (NT-proBNP) or self-reported functional capacity to clinical scores to estimate the risk of major adverse cardiac events (MACE).

**Design, Setting, and Participants:**

This cohort study included patients undergoing inpatient, elective, noncardiac surgery at 25 tertiary care hospitals in Europe between June 2017 and April 2020. Analysis was conducted in January 2023. Eligible patients were either aged 45 years or older with a Revised Cardiac Risk Index (RCRI) of 2 or higher or a National Surgical Quality Improvement Program, Risk Calculator for Myocardial Infarction and Cardiac (NSQIP MICA) above 1%, or they were aged 65 years or older and underwent intermediate or high-risk procedures.

**Exposures:**

Preoperative NT-proBNP and the following self-reported measures of functional capacity were the exposures: (1) questionnaire-estimated metabolic equivalents (METs), (2) ability to climb 1 floor, and (3) level of regular physical activity.

**Main Outcome and Measures:**

MACE was defined as a composite end point of in-hospital cardiovascular mortality, cardiac arrest, myocardial infarction, stroke, and congestive heart failure requiring transfer to a higher unit of care.

**Results:**

A total of 3731 eligible patients undergoing noncardiac surgery were analyzed; 3597 patients had complete data (1258 women [35.0%]; 1463 (40.7%) aged 75 years or older; 86 [2.4%] experienced a MACE). Discrimination of NT-proBNP or functional capacity measures added to clinical scores did not significantly differ (Area under the receiver operating curve: RCRI, age, and 4MET, 0.704; 95% CI, 0.646-0.763; RCRI, age, and 4MET plus floor climbing, 0.702; 95% CI, 0.645-0.760; RCRI, age, and 4MET plus physical activity, 0.724; 95% CI, 0.672-0.775; RCRI, age, and 4MET plus NT-proBNP, 0.736; 95% CI, 0.682-0.790). Benefit analysis favored NT-proBNP at a threshold of 5% or below, ie, if true positives were valued 20 times or more compared with false positives. The findings were similar for NSQIP MICA as baseline clinical scores.

**Conclusions and relevance:**

In this cohort study of nearly 3600 patients with elevated cardiovascular risk undergoing noncardiac surgery, there was no conclusive evidence of a difference between a NT-proBNP–based and a self-reported functional capacity–based estimate of MACE risk.

**Trial Registration:**

ClinicalTrials.gov Identifier: NCT03016936

## Key Points

**Question:**

Does the addition of N-terminal pro–B-type natriuretic peptide (NT-proBNP) to clinical risk scores provide a better estimate of risk of major adverse cardiac events (MACE) among patients undergoing noncardiac surgery than the addition of self-reported functional capacity measures?

**Findings:**

In this cohort study including 3597 patients, discrimination for MACE with the addition of NT-proBNP to clinical scores did not significantly differ when compared with self-reported functional capacity. Benefit analysis favored NT-proBNP if true positives were valued at least 20 times more than false positives.

**Meaning:**

The results of this study raise concerns about the clinical applicability of a NT-proBNP–based preoperative assessment.

## Teaser

This cohort study surgery compares the addition of N-terminal pro–brain natriuretic peptide measures vs self-reported functional capacity to clinical scores estimating the risk of major adverse cardiovascular events among patients undergoing noncardiac surgery.

## Introduction

For North America, the estimated need for surgical procedures amounts to 4647 per 100 000 individuals per year or 15.8 million operations annually.^1^ With over 4 million deaths worldwide every year,^2^ postoperative mortality represents a major population health problem. Major noncardiac surgery is associated with significant cardiovascular morbidity^3^ and the attributable fraction for 30-day mortality of myocardial injury after noncardiac surgery was estimated at 16%.^4^

To inform patients as a basis for shared decision-making and to tailor perioperative management to expected risk, clinicians need to estimate the probability of adverse events. National and international guidelines^5,6,7^ all consider the extent of the planned procedure and cardiovascular history for preoperative evaluation. As an additional crucial factor of risk estimation, relevant guidelines suggest different approaches. While American guidelines^6^ rely on functional capacity expressed in metabolic equivalents (either measured during cardiopulmonary exercise test or self-reported), Canadian guidelines^5^ rely on B-type natriuretic peptide (BNP) measurement. European guidelines^7^ suggest either self-reported functional capacity expressed by the ability to climb stairs or Duke Activity Status Index (DASI) or on BNP (class IIa).

The association between preoperative BNP concentrations and cardiovascular events after noncardiac surgery has been extensively explored.^8,9,10,11^ By the addition of the N-terminal pro-Brain Natriuretic Peptide (NT-proBNP) to the Revised Cardiac Risk Index (RCRI), the discrimination gain, expressed as the difference in area under the curve of receiver operating curve (ROC AUC) for major cardiac events (MACE) 30 days postsurgery ranged between 0.02 and 0.22. The corresponding value for 30-day all-cause mortality and MACE ranged between 0.06 and 0.07.^11^

Data directly comparing BNP and functional capacity are exceedingly rare. In the Measurement of Exercise Tolerance before Surgery Study, measures of functional capacity were assessed along with NT-proBNP (1347 participants).^12^ The discrimination (ROC AUC) for a composite of 30-day mortality and myocardial infarction was 0.67 for the RCRI plus DASI and 0.65 for the RCRI plus NT-proBNP (95% CI not reported). As such, while preoperative BNP concentrations appear to improve the projection of cardiovascular events after noncardiac surgery over clinical scores,^11^ their value compared with self-reported measures of functional capacity is not established.

The main objective of the MET: Reevaluation for Perioperative Cardiac Risk (MET-REPAIR)–NT-proBNP substudy was to compare the discrimination for MACE of models including NT-proBNP and validated clinical scores (Revised Cardiac Risk Index [RCRI]^13^ and National Surgical Quality Improvement Program, Risk Calculator for Myocardial Infarction and Cardiac [NSQIP MICA],^14^ respectively) with models that included self-reported functional capacity and those clinical scores. A secondary objective was to examine if estimates were improved by the addition of NT-proBNP to models including clinical scores and self-reported functional capacity measures.

## Methods

### Study Design and Setting

The MET-REPAIR NT-proBNP subcohort is a cohort study nested in MET-REPAIR, an international multicenter prospective cohort study.^15^ At the beginning of the study, centers could opt for participation in the subcohort. Enrollment occurred in 25 centers in 10 European countries between June 2017 and April 2020.

The study was carried out in accordance with the published research plan and the principles enunciated in the Declaration of Helsinki^16^ and the ICH-GCP Guidelines E6(R2).^17^ Prior to study initiation, local or national principal investigators (PI), as applicable, obtained approval from the responsible ethical board. All patients were informed and consented in writing using a dedicated METREPAIR–NT-proBNP informed consent form prior to enrollment. The project office and the principal PI trained the national PIs via teleconference on all aspects of the study and provided written definitions and data entry manuals to the centers. National PIs trained local PIs. This report follows the Strengthening the Reporting of Observational Studies in Epidemiology (STROBE) reporting recommendations (eMethods in Supplement 1).

### Study Population

Consecutive, consenting patients scheduled for inpatient noncardiac surgery were eligible if they were either (1) aged 45 years or older and undergoing elective elevated-risk noncardiac surgery as defined by either a RCRI of 2 or higher^13^ or a NSQIP MICA above 1%^14^; or (2) aged 65 years or older and undergoing elective intermediate or high-risk procedures.^18^ The exclusion criteria were: nonelective surgery (ie, within 72 hours of diagnosis), acute coronary syndrome or uncontrolled congestive heart failure within 30 days or stroke within 7 days of the planned day of surgery, outpatient surgery (ie, no overnight inpatient stay planned), patients unable to ambulate due to congenital or longstanding conditions, inability to complete the functional capacity questionnaire (language or literacy barriers), inability to consent or unwillingness to participate, and previous enrollment.

### Definition and Assessment of End Points

The primary end point was major adverse cardiac events (MACE), a composite end point of intra- or postoperative in-hospital cardiovascular mortality, nonfatal cardiac arrest, acute myocardial infarction (MI),^19^ stroke, and congestive heart failure requiring transfer to a higher unit of care or prolonging stay on intensive care unit or intermediate care (lasting 24 hours or longer). Study outcomes were adjudicated by the local PI based on standardized definitions after reviewing in-hospital records and any other relevant documentation obtained during the 30-day follow-up (eMethods in Supplement 1).^3,19^ Patients were followed-up until 30 days after surgery by phone or by mail. In case of events, relevant documents were requested from hospitals or general practitioners.

### Main Explanatory Variables

Preoperative NT-proBNP concentration was the main explanatory variable. NT-proBNP was sampled no longer than 30 days prior to surgery and analyzed locally using an NT-proBNP immune assay (Roche Diagnostics). The decision to conduct the measurements daily or in batches was at the discretion of the local PI and local laboratory. In the case of sample storage, storage temperature at –20 °C was recommended. Preoperative NT-proBNP concentrations were categorized according to the cut-off concentrations published by Duceppe et al^8^ as below 100 pg/mL, 100 to 200 pg/mL, 200 to 1500 pg/mL, and above 1500 pg/mL (to convert to nanograms per liter, multiply by 1.0).

### Self-Reported Measures of Functional Capacity

Self-reported measures of functional capacity consisted of functional capacity in METs (estimated using a 10-item questionnaire), ability to climb 1 floor, and level of regular physical activity.^20^ Patients completed the questionnaire^20^ no more than 30 days before surgery. If the operation was postponed for more than 30 days, patients were asked to complete the questionnaire again. Self-reported measures of functional capacity were categorized a priori. Self-reported METs were dichotomized at the cut-off endorsed by the relevant American guidelines^6^ (under 4 METs). The ability to climb 1 floor was categorized to mimic the recommendation of the ESC^7^ (less than 1 floor corresponding to less than 2 flights of stairs), and the self-reported level of regular physical activity was categorized as established in the METREPAIR parent sample (inactive defined as 20 minutes of activity per week or less).^15^

### Covariables

Other independent variables were the RCRI and age in the primary analysis and the NSQIP MICA in a planned additional analysis (age is part of the NSQIP MICA and was therefore not added). Race and ethnicity were not considered as covariables because they have not been shown to be associated with the primary outcome in previous studies. Both clinical risk scores are validated and commonly used for cardiovascular risk estimation prior to noncardiac surgery.^6,8,11,21^

### Statistical Analysis

For this nested cohort, we aimed for 3500 patients, corresponding to 70 composite events assuming an incidence of 2% for the primary composite end point.^22,23,24,25^ The estimated number of events was expected to allow adjustment with up to 6 to 7 covariates.^26^

Baseline characteristics and clinical outcomes were summarized as counts with percentages. For comparisons of outcomes, we used mixed-effects logistic regression to model binary clinical outcomes where the log odds of the outcome were modeled as a linear function of a mixture of fixed effects of the independent variables and a random effect by country. Model performance was evaluated using the area under the curve (AUC) of the receiver operation characteristics (ROC) curve and the Brier score. We tested for AUC equality between models including RCRI, age, and NT-proBNP vs RCRCI, age, and functional capacity measures using the DeLong test for 2 correlated ROC curves.^27^ During the review process, DeLong tests were additionally calculated for RCRI plus age vs RCRI, age, and NT-proBNP and for RCRI plus age vs RCRI, age, and functional capacity measures. The same applied for NSQIP MICA-based models. Each model’s apparent performance was assessed in the studied data set. This allowed us to quantify performance for model comparison. We did not correct model performance for optimism with bootstrapping since the aim was model comparison only and not model validation.

In addition, we compared the models using net benefit approaches^28,29,30^: we plotted net benefit at misclassification cost ranging from 0 to 15% (decision curves). A misclassification cost of 5% (1 in 20) corresponded to the acceptance of 20 false positives for 1 true positive; a misclassification cost of 15% (1 in 6.7) is the acceptance of 6.7 false positives for 1 true positive. We calculated weighted comparison (WC = change in sensitivity + [(1 − prevalence/prevalence) × relative cost (FP/TP) × change in specificity]) at the observed incidence for each in-hospital MACE and 30-day MACE and a misclassification cost predefined at 10%, ie, a trade-off at 10 false positives per 1 true positive, corresponding to a 10-fold higher relative weighting of true positive over false positive.^28^ To aid interpretation, we converted WC to net benefit equivalent (incremental number of true positives per 1000 patients).^30^ We conducted a full-case analysis. We applied 2-sided level of significance at *P* < .05.

All statistical analyses were performed using R version 4.1.2 (R Project for Statistical Computing). Mixed-effects logistic models were fitted using the glmer function of the R statistical package lme4. The analysis was conducted in January 2023.

## Results

### Descriptive Data

Among 3597 of 3731 patients with complete data, 1258 (35.0%) were women and 1463 (40.7%) were aged 75 years or older (Table 1). Baseline characteristics of METREPAIR patients eligible for the NT-proBNP substudy who declined participation or were not captured are reported in eTable 1 in Supplement 1. Of 3597 patients, 86 developed in-hospital MACE (2.4%) (Figure). These consisted of 23 (0.6%) cardiac deaths, 27 (0.8%) myocardial infarctions, 16 (0.4%) nonfatal cardiac arrests, 17 (0.5%) heart failures requiring transfer to a higher unit of care or prolonged stay in intensive care unit (ICU) or intermediate care (ie, 24 hours or longer), and 9 (0.3%) strokes. At 30 days, 103 of 3593 patients (2.9%) had suffered a MACE. Six patients (0.2%) had incomplete answers to the METs questions. Therefore, the analyses on METs based on 86 in-hospital MACE in 3591 patients and 103 30-day MACE in 3587 patients. NT-proBNP was sampled within 1 day prior to surgery in 3119 patients (86.7%) and 7 days prior to surgery in 3475 (96.6%) of the patients.

**Table 1.** Baseline Characteristics of the Whole Cohort and by In-Hospital MACE

Characteristics	Participants, No. (%)		
	All (n = 3597)	MACE (n = 86)	No MACE (n = 3511)
Age, y			
45-74	2134 (59.3)	37 (43.0)	2097 (59.7)
≥75	1463 (40.7)	49 (57.0)	1414 (40.3)
Sex			
Female	1258 (35.0)	27 (31.4)	1231 (35.1)
Male	2339 (65)	59 (68.6)	2280 (64.9)
RCRI			
Low (≤1 point)	1474 (41.0)	22 (25.6)	1452 (41.4)
Moderate (2 points)	1343 (37.3)	33 (38.4)	1310 (37.3)
High (≥3 points)	780 (21.7)	31 (36.0)	749 (21.3)
NSQIP-MICA risk, %			
Mean (SD)	1.84 (1.77)	2.31 (1.94)	1.83 (1.76)
Median (IQR)	1.34 (0.58-2.46)	2.08 (0.78-3.23)	1.33 (0.58-2.46)
Range	0.02-15.06	0.10-11.61	0.02-15.06
NT-proBNP, pg/mL			
<100	1131 (31.5)	15 (17.4)	1116 (31.8)
100-200	825 (22.9)	11 (12.8)	814 (23.2)
200-1500	1320 (36.7)	38 (44.2)	1282 (36.5)
≥1500	321 (8.9)	22 (25.6)	299 (8.5)
Self-reported functional capacity			
<4 METs^a^	454 (12.6)	19 (21.1)	435 (12.4)
Stair climbing <1 floor	402 (11.2)	15 (17.4)	387 (11.0)
Inactive or regular physical activity ≤20 min/wk activity^b^	2216 (61.6)	67 (78.0)	2194 (62.5)

Abbreviations: MACE, major adverse cardiac events; MET, metabolic equivalent; NT-proBNP, N-terminal pro–brain natriuretic peptide; NSQIP MICA, National Surgical Quality Improvement Program, Risk calculator for Myocardial Infarction and Cardiac; RCRI, Revised Cardiac Risk Index.

^a^ Six of 3597 patients had incomplete answers to the METs questions; the analyses on METs based on 86 in-hospital MACE in 3591 patients and 103 MACE in 3587 patients.

^b^ Referring to brisk walking, jogging or running, cycling, swimming, or vigorous sports at a comfortable pace or other activities requiring similar levels of exertion; MACE is defined as a composite of intra- or postoperative in-hospital cardiovascular mortality, nonfatal cardiac arrest, acute myocardial infarction, stroke, and congestive heart failure requiring transfer to a higher unit of care or prolonging stay on intensive care unit or intermediate care 24 hours or longer.

**Figure.**
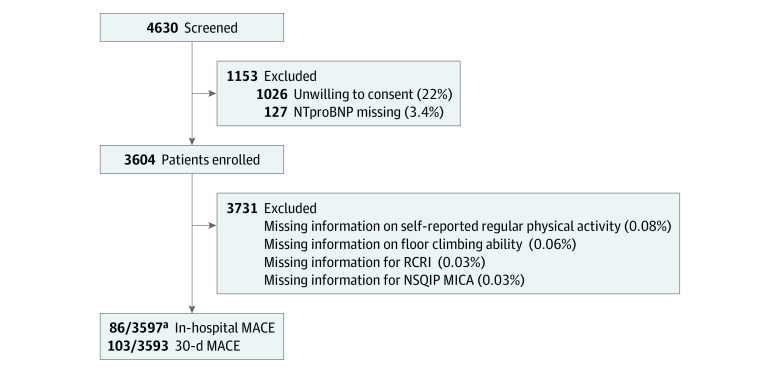
Study Flowchart MACE indicates major adverse cardiac events; NSQIP MICA, National Surgical Quality Improvement Program, Risk calculator for Myocardial Infarction and Cardiac; NT-proBNP, N-terminal pro–brain natriuretic peptide; RCRI, Revised Cardiac Risk Index. ^a^Six additional patients had incomplete answers to the METs questions; the analyses on METs based on 86 in-hospital MACE in 3591 patients and 103 MACE in 3587 patients.

### Estimated Risk of MACE Occurrence Using RCRI Plus NT-proBNP vs RCRI Plus Self-Reported Functional Capacity

The addition of NT-proBNP to a model of RCRI plus age significantly increased discrimination for in-hospital MACE ROC AUC (0.736; 95% CI, 0.682-0.790; *P* = .03) but not for 30-day MACE (0.721; 95% CI, 0.670-0.771; *P* = .39) (Table 2) The discrimination gain by the addition of functional capacity measures to RCRI plus age was not significant (Table 2). The addition of NT-proBNP to the RCRI plus age measure led to the numerically higher ROC AUC than the addition of any of the self-reported measures of functional capacity (fewer than 4 METs, less than 1 floor of stairs, and less than 20 min/wk regular physical activity). This applied for each in-hospital and for 30-day MACE (Table 2). However, the discrimination of the model adding NT-proBNP did not significantly differ from the models adding functional capacity measures to RCRI and age (Table 2). Of note, in contrast to the cut-off implemented in the parent METREPAIR study (1 floor or less),^15^ the ability to climb stairs dichotomized at 1 floor as suggested by the ESC guidelines (less than 1 floor corresponding to less than 2 flights of stairs)^7^ was independently associated with 30-MACE but not with in-hospital MACE after adjustment by RCRI plus age (eTable 2 in Supplement 1).

**Table 2.** Results for In-Hospital and 30-Day MACE From Mixed Effect Logistic Regression Models Based on Revised Cardiac Risk Index (RCRI)^a^

Variable	In-hospital MACE			30-d MACE		
	Brier score	ROC AUC (95% CI)	*P* value to NT-proBNP model	Brier score	ROC AUC (95% CI)	*P* value to NT-proBNP model
RCRI + age	0.023	0.694 (0.636-0.753)	NA	0.023	0.687 (0.628-0.745)	NA
RCRI, age, and NTproBNP	0.023	0.736 (0.682-0.790)	NA	0.027	0.721 (0.670-0.771)	NA
RCRI, age, <4 METs^b^	0.023	0.704 (0.646-0.763)	.12	0.027	0.681 (0.625-0.736)	.06
RCRI, age, and <1 floor	0.023	0.702 (0.645-0.760)	.08	0.027	0.685 (0.632-0.738)	.07
RCRI, age, and <20 min/w, regular physical activity^c^	0.023	0.724 (0.672-0.775)	.56	0.027	0.686 (0.634-0.739)	.10

Abbreviations: AUC, area under the receiving operator curve; MACE, major adverse cardiac events; METs, metabolic equivalents; NA, not applicable; NT-proBNP, N-terminal pro–brain natriuretic peptide; RCRI, Revised Cardiac Risk Index.

^a^ De Long tests for comparisons: RCRI, age, and NT-proBNP vs RCRI plus age, *P* = .03 for in-hospital MACE, *P* = .39 for 30-day MACE; RCRI, age, and 4 METs vs RCRI plus age, *P* = .35 for in-hospital MACE, *P* = .45 for 30-day MACE; RCRI, age, and stair climbing vs RCRI plus age, *P* = .23 for in-hospital MACE, *P* = .28; RCRI, age, physical activity vs RCRI plus age, *P* = .07 for in-hospital MACE, *P* = .32 for 30-day MACE.

^b^ Six of 3597 patients had incomplete answers to the METs questions; the analyses on METs based on 86 in-hospital MACE in 3591 patients and 103 MACE in 3587 patients.

^c^ Regular physical activity to brisk walking, jogging or running, cycling, swimming, or vigorous sports at a comfortable pace or other activities requiring similar levels of exertion.

The addition of NT-proBNP to 4 METs and to the regular physical activity level, respectively, improved discrimination for in-hospital MACE (ROC AUC: RCRI, age, 4MET, and NT-proBNP, 0.741; 95% CI, 0.688-0.795 vs RCRI, age, and 4MET, 0.704; 95% CI, 0.646-0.763; *P* = .03; RCRI, age, physical activity, and NT-proBNP, 0.750; 95% CI, 0.699-0.801 vs RCRI, age, and physical activity, 0.724; 95% CI, 0.672-0.775; *P* = .04) (Table 2; eTable 3 in Supplement 1). The addition of NT-proBNP to the ability to climb stairs did not significantly improve discrimination for in-hospital MACE compared with a model based only on stair climbing. Brier scores were similar for all models (eTable 3 in Supplement 1). The addition of NT-proBNP to each self-reported measure of functional capacity significantly improved discrimination for 30-day MACE; however, the effect size was limited (eTable 3 in Supplement 1).

Decision curves suggested that the net benefit of models including NT-proBNP was largest for misclassification cost of 5% or less, ie, at trade-offs in the order of 20 or more false positives per 1 true positive (or relative weighting of 20 or more for true positive over false positive) (eFigures 1 and 2 in Supplement 1). At the incidence of in-hospital MACE of 2.4% and of 30-day MACE of 2.9%, respectively, and at a misclassification cost of 10% (ie, acceptance of 10 false positives per 1 true positive), benefit equivalent for models including NT-proBNP was marginal (depending on the model in the order of 1 to 10 incremental true positive patients per 1000 patients) (Table 3; eTable 4 in Supplement 1).

**Table 3.** Weighted Comparison and Benefit Equivalent for In-Hospital MACE for Revised Cardiac Risk Index (RCRI)-Based Models^a^

Comparison	In-hospital MACE		30-d MACE	
	Weighted comparison	Benefit equivalent (per 1000 patients)	Weighted comparison	Benefit equivalent (per 1000 patients)
RCRI, age, and NT-proBNP vs RCRI, age, and <4 MET^b^	0.134	3	0.209	6
RCRI, age, and NT-proBNP vs RCRI, age, and <1 floor of stairs	0.270	6	0.241	7
RCRI, age, and NT-proBNP vs RCRI, age, and level of physical activity^c^	0.269	6	0.331	10

Abbreviations: METs, metabolic equivalents; NT-proBNP, N-terminal pro–brain natriuretic peptide; RCRI, revised cardiac risk index.

^a^ In-hospital MACE incidence was 2.4%, 30-day MACE incidence was 2.9%, and misclassification cost was set at 10%.

^b^ Six of 3597 patients had incomplete answers to the METs questions; the analyses on METs based on 86 in-hospital MACE in 3591 patients and 103 MACE in 3587 patients.

### Projection of MACE Occurrence Using NSQIP MICA Plus NT-proBNP vs NSQIP MICA Plus Self-Reported Functional Capacity

The addition of NTPproBNP to the NSQIP-MICA significantly improved discrimination for in-hospital MACE (ROC AUC, 0.732; 95% CI, 0.681-0.783; *P* = .04) but not for 30-day MACE (DeLong test, *P* = .19) (Table 4). The discrimination gain by the addition of functional capacity measures to NSQIP MICA was not significant. The addition of NT-proBNP to the NSQIP MICA led to the numerically higher ROC AUC than the addition of any of the self-reported measures of functional capacity. This applied for each in-hospital and for 30-day MACE (Table 4). However, the discrimination of the model adding NT-proBNP did not significantly differ from the models adding functional capacity measures to NSQIP MICA (Table 4).

**Table 4.** Results for In-Hospital and 30-Day MACE From Mixed Effect Logistic Regression Models Based on NSQIP MICA^a^

Variables	In-hospital MACE			30-d MACE		
	Brier Score	ROC AUC (95% CI)	*P* value to NT-proBNP model	Brier Score	ROC AUC (95% CI)	*P* value to NT-proBNP model
NSQIP MICA	0.023	0.676 (0.623-0.728)	NA	0.023	0.666 (0.614-0.719)	NA
NSQIP MICA + NT-proBNP	0.023	0.732 (0.681-0.783)	NA	0.027	0.715 (0.665-0.765)	NA
NSQIP MICA + <4 METs^b^	0.023	0.690 (0.636-0.744)	.14	0.028	0.668 (0.616-0.719)	.10
NSQIP MICA + <1 floor stairs	0.023	0.684 (0.633-0.735)	.07	0.028	0.671 (0.622-0.719)	.09
NSQIP MICA + <20 min/wk regular physical activity^c^	0.023	0.705 (0.655-0.754)	.07	0.028	0.674 (0.626-0.721)	.14

Abbreviations: ROC AUC, area under the receiver operating curve; MACE, major adverse cardiac events; METs, metabolic equivalents; NA, not applicable; NT-proBNP, N-terminal pro–brain natriuretic peptide; NSQIP MICA, National Surgical Quality Improvement Program, Risk Calculator for Myocardial Infarction and Cardiac Arrest.

^a^ All models include a random intercept by country. DeLong tests for comparisons: NT-proBNP plus NSQIP MICA vs NSQIP MICA, *P* = .04 for in-hospital MACE, *P* = .19 for 30-day MACE; NSQIP MICA plus 4 METs vs NSQIP MICA, *P* = .35 for in-hospital MACE, *P* = .48 for 30-day MACE; NSQIP MICA plus stair climbing vs NSQIP MICA, *P* = .48 for in-hospital MACE, *P* = .40 for 30-day MACE; NSQIP MICA plus physical activity vs NSQIP MICA, *P* = .19 for in-hospital MACE, *P* = .38 for 30-day MACE.

^b^ Six of 3597 patients had incomplete answers to the METs questions; the analyses on METs based on 86 in-hospital MACE in 3591 patients and 103 MACE in 3587 patients.

^c^ Regular physical activity to brisk walking, jogging or running, cycling, swimming, or vigorous sports at a comfortable pace or other activities requiring similar levels of exertion.

Corresponding adjusted ORs are reported in eTable 5 in Supplement 1; of note, in contrast to the cut-off implemented in the parent METREPAIR study,^15^ the ability to climb stairs dichotomized at 1 floor or less as suggested by the ESC guidelines^7^ was independently associated with 30-MACE but not with in-hospital MACE after adjustment by NSQIP MICA.

The addition of NT-proBNP to 4 METs (plus NSQIP-MICA) resulted in a numerically larger ROC AUC but it missed significance (ROC AUC, 0.737 vs 0.690; *P* = .05). The addition of NT-proBNP to stair climbing and to the self-reported level of physical activity improved discrimination (ROC AUC: NSQIP-MICA, stair climbing, and NT-proBNP, 0.734; 95% CI, 0.684-0.784 vs NSQIP-MICA plus stair climbing, 0.684; 95% CI, 0.633-0.754; *P* = .04; NSQIP-MICA, physical activity, and NT-proBNP, 0.742; 95% CI, 0.691-0.793 vs NSQIP-MICA plus physical activity, 0.705; 95% CI, 0.655-0.754; *P* = .05). Brier scores were similar for all models. The addition of NT-proBNP to self-reported measures of functional capacity improved discrimination for 30-day MACE (eTable 6 in Supplement 1).

Decision curves suggested that the net benefit of NSQIP-MICA-based models including NT-proBNP was largest for misclassification cost of 5% or below, ie, at trade-offs in the order 20 or more false positives per each true positive (or relative weighting of true positive to false positive of 20 or more) (eFigures 3 and 4 in Supplement 1). At the incidence of in-hospital MACE of 2.4% and at a misclassification cost of 10% (ie, trade-off at 10 false positives per 1 true positive) benefit equivalent for NSQIP-MICA-based models including NT-proBNP were marginal (depending on the model in the order of 0 to 9 more true positive patients per 1000 patients) (eTable 7 in Supplement 1).

## Discussion

### Main Findings

After adjusting for RCRI and age, each of self-reported MET below 4, inability to climb 1 floor, inactivity or limited regular physical activity, and NT-proBNP were independently associated with 30-day MACE. The same findings applied to adjustment using NSQIP MICA.

Albeit numerically larger, discrimination for either in-hospital or 30-day MACE of models using NT-proBNP did not reach significant difference compared with models using self-reported measures of functional capacity. This applied both for models using RCRI and age and those using NSQIP MICA as the baseline. Discrimination for 30-day MACE significantly improved by the addition of NT-proBNP to functional capacity measures (plus clinical risk score). A similar pattern was seen for in-hospital MACE, but it did not consistently reach significance.

Decision analysis suggested that models using NT-proBNP provided a net benefit over models using functional capacity measures at lower threshold probabilities, ie, if 20 or more false positives were considered an acceptable trade-off per 1 true positive. At the predefined threshold of 10% (trade-off at 10 false positives per 1 true positive), benefit by the use of NT-proBNP over self-reported functional capacity measures for projection of perioperative MACE was marginal.

As such, there was no conclusive evidence of a difference between a NT-proBNP–based and a self-reported functional capacity–based MACE projection. While a difference may still exist, its size appears to be in an order of magnitude such as not to be conclusively detected within a sample of near to 3600 patients at elevated cardiovascular risk. In our opinion, this raises concerns regarding the clinical relevance of the potential difference and therefore regarding the clinical applicability of a NT-proBNP–based preoperative risk assessment.

### Comparison With Previous Studies

In line with previous studies,^8,9,10,11^ NT-proBNP was significantly associated with MACE after noncardiac surgery. This confirms the well-established role of NT-proBNP as prognostic factor, ie, as factor influencing the risk of adverse outcomes.

In terms of projection, the evidence appears more nuanced. In the largest sample to date,^8^ the addition of NT-proBNP to the RCRI resulted in a ROC AUC of 0.73 (95% CI, 0.72-0.74) for a composite of cardiovascular death and myocardial injury and of 0.75 (95% CI, 0.73-0.78) for all-cause mortality and myocardial infarction. In addition to confirming the moderate ROC AUC (0.73; 95% CI, 0.68-0.79) for in-hospital MACE for RCRI plus NT-proBNP previously described by Duceppe et al,^8^ in the present cohort we report discrimination in the same order of magnitude when NT-proBNP was added to the NSQIP MICA, another commonly used clinical risk score.^6,18^ In the Measurement of Exercise Tolerance before Surgery study (including 1347 participants),^12^ the discrimination (ROC AUC) for a composite of 30-day mortality and myocardial infarction was 0.65 for the RCRI plus NT-proBNP. NT-proBNP did not improve net reclassification. For the composite end point of 30-day mortality and myocardial injury, ROC AUC amounted to 0.71 for the addition of NT-proBNP to age, sex, and RCRI. While the assessment of model performance is not limited to discrimination, the body of evidence including other measures is less conspicuous.^11^

Of note, the focus of this analysis was not the quantification of the predictive value of NT-proBNP per se; rather, it specifically aimed at the direct comparison between NT-proBNP and self-reported functional capacity measures, an easily implementable, low-cost approach commonly used in clinical practice. The evidence in this regard is scarce. Duceppe at al^8^ did not report on functional capacity. In the Measurement of Exercise Tolerance before Surgery study, ^12^ the discrimination for a composite of 30-day mortality and myocardial infarction was 0.67 for the RCRI plus DASI, ie, a similar order as for NT-proBNP (0.65). For the composite end point of 30-day mortality and myocardial injury, ROC AUC amounted to 0.71 for the addition of DASI to age, sex, and RCRI, again in the same order as for NT-proBNP (0.71). Formal testing for AUC difference between the baseline model with DASI vs with continuous NT-proBNP was not reported.^12^ In the present cohort, while discrimination was numerically higher for models using NT-proBNP added to the clinical risk score, it did not reach a significant difference compared with self-reported functional capacity measures, as supported by wide overlap in 95% CI intervals. Of note, the limited potential benefit of NT-proBNP measurement over self-reported measured of functional capacity for MACE projection is supported by the findings of benefit analysis suggesting that NT-proBNP–based assessment is preferable if 20 or more false positives are acceptable to detect 1 additional true positive.

In a secondary analysis of the Measurement of Exercise Tolerance before Surgery study,^31^ the ROC AUC for the model using both functional capacity (DASI) and NT-proBNP was 0.66. Discrimination improvement compared with a model with DASI only was not reported. In our data, the combined use of both functional capacity measures and NT-proBNP improved discrimination for 30-day MACE over clinical risk scores. As such self-reported functional measures and NT-proBNP appear to provide complementary information. However, benefit analyses suggested that the benefit of the combined use is limited (less than 10 additional true positives per 1000 patients assuming a trade-off at 10%).

### Strengths and Limitations

The analytical approach was strengthened first by the joint assessment of both self-reported measures and NT-proBNP in a large prospective cohort. This allowed for the comparison of risk assessment approaches endorsed by relevant guidelines, ie, the consideration (in addition to clinical factors) of functional capacity in the American and European guidelines^6,7^ and of B-type natriuretic peptide in the Canadian guidelines.^5^ We also assessed the combined use of self-reported functional capacity measures and NT-proBNP. Second, the evaluation was not limited to RCRI-based models but also included the evaluation of models based on the NSQIP MICA. Finally, in addition to the quantification of independent associations and of measures of model performance, we applied a decision analytical approach^28,29,30^ as recommended.^32,33^

This study had several limitations. First, the use of a composite end point (MACE) was mainly driven by cardiac death and myocardial infarction. The preferred outcome according to the Standardized Endpoints in Perioperative Medicine (StEP) initiative that was published after conclusion of data collection, is all-cause death and myocardial infarction.^34^ While we did not conduct a formal analysis, because less than 20% of the registered events were heart failures, we consider that exclusion of heart failure from the MACE composite would not relevantly affect the findings of the limited gain of NT-proBNP compared with functional capacity. If anything, considering the closer link between NT-proBNP and heart failure than with death and myocardial infarction, one would expect the discrimination gain using NT-proBNP for these end points to be even more limited. Second, we did not conduct systematic postoperative troponin surveillance with the resulting risk of misclassification. However, the discrimination we described for NT-proBNP (ROC AUC, 0.74) was essentially the same as the discrimination described by Duceppe et al^8^ for vascular death and myocardial injury (ROC AUC, 0.73) and for all-cause mortality at myocardial infarction (ROC AUC, 0.75).^8^

Third, while our primary end point was in-hospital MACE, our analysis applied an NT-proBNP cut-off developed for vascular mortality and myocardial injury after noncardiac surgery.^8^ However, those cut-offs demonstrated significant associations also for other end points in the publication by Duceppe and colleagues.^8^ Fourth, the protocol did not mandate the masking of clinical care professionals for NT-proBNP concentrations, so that we cannot exclude performance bias as a factor to some extent. Fifth, we did not conduct correction for multiple testing. As such, we consider that cautious appraisal of *P* values is warranted and we advise against the interpretation of De Long *P* values slightly exceeding 0.05 as tendencies hinting at differences between NT-proBNP and self-reported measures. Finally, while the sample included almost 3600 patients, the number of events (86 in-hospital and 103 30-day MACE) was not sufficient to provide conclusive evidence of any difference between a NT-proBNP–based and a self-reported functional capacity-based MACE projection. In our opinion, this suggests that the size of the potential difference between the 2 approaches may not be of clinical relevance.

## Conclusions

In this cohort study of approximately 3600 patients with elevated cardiovascular risk undergoing noncardiac surgery, there was no conclusive evidence of a difference between a NT-proBNP–based and a self-reported functional capacity–based MACE projection. Our results suggest that caution about the clinical applicability of a NT-proBNP–based preoperative assessment is warranted.

## Notes

Supplement 1.
eMethods.
**eTable 1.** Baseline Characteristics of Patients Included and Excluded From the NT-proBNP Substudy **eTable 2.** Adjusted Odds Ratios (OR) for In-Hospital and 30-day MACE for RCRI-Based Models Including NT-proBNP or Each of the Assessed Self-Reported Functional Capacity Measures **eTable 3.** Brier Score and ROC AUC for In-Hospital and 30-day MACE From Mixed Effect Logistic Regression Models Based the Addition of NT-proBNP to Models Including RCRI, Age, and Functional Capacity Measures **eTable 4.** Weighted Comparison and Benefit Equivalent for MACE for the Addition of NT-proBNP to Models Including RCRI, Age, Functional Capacity Measures **eTable 5.** Adjusted Odds Ratios (OR) for In-Hospital and 30-day MACE for NSQIP MICA-Based Models Including NT-proBNP or Each of the Assessed Self-Reported Functional Capacity Measures **eTable 6.** Brier Score and ROC AUC for MACE From Mixed Effect Logistic Regression Models Based on for the Addition of NT-proBNP to Models Including NSQIP MICA Plus Functional Capacity Measures **eTable 7.** Weighted Comparison and Benefit Equivalent for In-Hospital and for 30-day MACE for NSQIP-MICA-Based Models **eFigure 1.** Decision Curves of Various RCRI-Based Models for In-Hospital MACE **eFigure 2.** Decision Curves of Various RCRI-Based Models Combining NT-proBNP and Self-Reported Functional Capacity Measures for In-Hospital MACE **eFigure 3.** Decision Curves of Various NSQIP MICA-Based Models for In-Hospital MACE **eFigure 4.** Decision Curves of Various NSQIP MICA-Based Models Combining NT-proBNP and Self-Reported Functional Capacity Measures for In-Hospital MACE

Supplement 2.
Nonauthor Collaborators

Supplement 3.
Data Sharing Statement
