# Supplementary material for: NT-proBNP or Self-Reported Functional Capacity in Estimating Risk of Cardiovascular Events After Noncardiac Surgery
Source: JAMA Netw Open. 2023 Nov 8;6(11):e2342527. doi: 10.1001/jamanetworkopen.2023.42527 (PMC10632953; doi:10.1001/jamanetworkopen.2023.42527)
Supplement: Supplement 1. — eMethods. eTable 1. Baseline Characteristics of Patients Included and Excluded From the NT-proBNP Substudy eTable 2. Adjusted Odds Ratios (OR) for In-Hospital and 30-day MACE for RCRI-Based Models Including NT-proBNP or Each of the Assessed Self-Reported Functional Capacity Measures eTable 3. Brier Score and ROC AUC for In-Hospital and 30-day MACE From Mixed Effect Logistic Regression Models Based the Addition of NT-proBNP to Models Including RCRI, Age, and Functional Capacity Measures eTable 4. Weighted Comparison and Benefit Equivalent for MACE for the Addition of NT-proBNP to Models Including RCRI, Age, Functional Capacity Measures eTable 5. Adjusted Odds Ratios (OR) for In-Hospital and 30-day MACE for NSQIP MICA-Based Models Including NT-proBNP or Each of the Assessed Self-Reported Functional Capacity Measures eTable 6. Brier Score and ROC AUC for MACE From Mixed Effect Logistic Regression Models Based on for the Addition of NT-proBNP to Models Including NSQIP MICA Plus Functional Capacity Measures eTable 7. Weighted Comparison and Benefit Equivalent for In-Hospital and for 30-day MACE for NSQIP-MICA-Based Models eFigure 1. Decision Curves of Various RCRI-Based Models for In-Hospital MACE eFigure 2. Decision Curves of Various RCRI-Based Models Combining NT-proBNP and Self-Reported Functional Capacity Measures for In-Hospital MACE eFigure 3. Decision Curves of Various NSQIP MICA-Based Models for In-Hospital MACE eFigure 4. Decision Curves of Various NSQIP MICA-Based Models Combining NT-proBNP and Self-Reported Functional Capacity Measures for In-Hospital MACE [file jamanetwopen-e2342527-s001.pdf]

Supplemental Online Content

Buse GL, Larmann J, Gillmann H, et al; and the METREPAIR NT-proBNP Subcohort Investigators. NT-proBNP or Self-Reported Functional Capacity in Estimating Risk of Cardiovascular Events After Noncardiac Surgery. *JAMA Netw Open.* 2023;6(11):e2342527. doi:10.1001/jamanetworkopen.2023.42527

eMethods.

eTable 1. Baseline characteristics of patients included and excluded from the NT-proBNP substudy

eTable 2. Adjusted Odds Ratios (OR) for In-Hospital and 30-day MACE for RCRI-Based Models Including NT-proBNP or Each of the Assessed Self-Reported Functional Capacity Measures

eTable 3. Brier Score and ROC AUC for In-Hospital and 30-day MACE From Mixed Effect Logistic Regression Models Based the Addition of NT-proBNP to Models Including RCRI, Age, and Functional Capacity Measures

eTable 4. Weighted Comparison and Benefit Equivalent for MACE for the Addition of NT-proBNP to Models Including RCRI, Age, Functional Capacity Measures

eTable 5. Adjusted Odds Ratios (OR) for In-Hospital and 30-day MACE for NSQIP MICA-Based Models Including NT-proBNP or Each of the Assessed Self-Reported Functional Capacity Measures

eTable 6. Brier Score and ROC AUC for MACE From Mixed Effect Logistic Regression Models Based on for the Addition of NT-proBNP to Models Including NSQIP MICA Plus Functional Capacity Measures

eTable 7. Weighted Comparison and Benefit Equivalent for In-Hospital and for 30-day MACE for NSQIP-MICA-Based Models

eFigure 1. Decision Curves of Various RCRI-Based Models for In-Hospital MACE

eFigure 2. Decision Curves of Various RCRI-Based Models Combining NT-proBNP and Self-Reported Functional Capacity Measures for In-Hospital MACE

eFigure 3. Decision Curves of Various NSQIP MICA-Based Models for In-Hospital MACE

eFigure 4. Decision Curves of Various NSQIP MICA-Based Models Combining NT-proBNP and Self-Reported Functional Capacity Measures for In-Hospital MACE

This supplemental material has been provided by the authors to give readers additional information about their work.

©2023 Buse GL et al. *JAMA Network Open.*

eMethods

1.1. <u>Outcomes Definitions</u>

All outcomes were assessed until discharge or up to 30 days after surgery if hospitalization lasted beyond 30 days. In several centers (148/150), additional follow-up by phone or mail was conducted at 30 days. Centers had to define prior to ethical board submission if they planned to conduct in-hospital follow-up only or additional follow-up at 30 days as well.

Cardiovascular mortality

Any death presumably of cardiovascular origin including deaths following myocardial infarction, cardiac arrest, stroke, heart failure or cardiogenic shock, complications of cardiac revascularization procedure, or death of unknown cause.

Non-fatal cardiac arrest

Successful resuscitation from documented or presumed ventricular fibrillation, sustained ventricular tachycardia, asystole, a pulseless electrical activity requiring cardiopulmonary resuscitation, pharmacological therapy, or cardiac defibrillation, or documented physician´s diagnosis of nonfatal cardiac arrest in the clinical records.

Acute myocardial infarction

According to the fourth universal definition, i.e., evidence of myocardial necrosis in a clinical setting consistent with acute myocardial ischemia. Under these conditions, any one of the following criteria meets the diagnosis for MI:

• Detection of a rise and/or fall of cardiac biomarker values [preferably cardiac troponin (cTn)] with at least one value above the 99th percentile upper reference limit (URL) and with at least one of the following:

- Symptoms of ischemia;

- New or presumed new significant STsegment elevation;

- T wave (ST–T) changes or new left bundle branch block;

- Development of pathological Q waves in the ECG;

- Imaging evidence of new loss of viable myocardium or new regional wall motion abnormality.

- Identification of an intracoronary thrombus by angiography or autopsy.

• Cardiac death with symptoms suggestive of myocardial ischemia and presumed new ischemic ECG changes or new left bundle branch block, but death occurred before cardiac biomarkers were obtained, or before cardiac biomarker values would be increased.

• Percutaneous coronary intervention (PCI) related MI is arbitrarily defined by the elevation of cTn values (>5 × 99th percentile URL) in patients with normal baseline values (≤99th

©2023 Buse GL et al. *JAMA Network Open.*

percentile URL) or a rise of cTn values >20% if the baseline values are elevated and are stable or falling. In addition, either (i) symptoms suggestive of myocardial ischemia or (ii) new ischemic ECG changes or (iii) angiographic findings consistent with a procedural complication, or (iv) imaging demonstration of new loss of viable myocardium or new regional wall motion abnormality are required.

• Stent thrombosis associated with MI when detected by coronary angiography or autopsy in the setting of myocardial ischemia and with a rise and/or fall of cardiac biomarker values with at least one value above the 99th percentile URL.

• Coronary artery bypass grafting (CABG) related MI is arbitrarily defined by the elevation of cardiac biomarker values (>10 × 99th percentile URL) in patients with normal baseline cTn values (≤99th percentile URL). In addition, either (i) new pathological Q waves or new LBBB,

or (ii) angiographic documented new graft or new native coronary artery occlusion, or (iii) imaging evidence of new loss of viable myocardium or new regional wall motion abnormality or documented physician´s diagnosis of myocardial infarction in the clinical records.

Congestive heart failure requiring transfer to a higher unit of care or prolonged stay in the ICU (≥24h)

The report of at least one clinical sign (i.e., elevated jugular venous pressure, respiratory rales/crackles, crepitations, or presence of S3) and one radiographic finding (i.e., vascular redistribution, interstitial/alveolar pulmonary edema) or the documented physician´s diagnosis of congestive heart failure AND clinical records indicating that heart failure was a main trigger for ICU or intermediate care transfer and for prolonged ICU stay, respectively. ICU or intermediate care unit refers to units with continuous monitoring of vitals, the possibility of noninvasive and/or invasive ventilation, and of administration of iv-inotropes and vasopressors. Prolonged refers to an extension of ≥ 24h of expected ICU/IMC duration after specific surgical procedures according to local standards.

Stroke

Report of new focal neurological deficit thought to be vascular in origin with signs and symptoms lasting > 24h or documented physician´s diagnosis of stroke in the clinical records.

©2023 Buse GL et al. *JAMA Network Open.*

eTable 1. Baseline characteristics of patients included and excluded from the NTproBNP substudy

	Included n=3604	Excluded n=1026
**MACE in-hospital**	86 (2.4%)	23 (2.2%)
**MACE within 30 days from surgery**	103 (2.9%)	31 (3.0%)
**Age (years)**		
40-74	2137 (59.3%)	573 (55.8%)
>=75	1467 (40.7%)	453 (44.2%)
**Sex**		
Male	2344 (65.0%)	617 (60.1%)
Female	1260 (35.0%)	409 (39.9%)
**NTproBNT (pg/ml)**		
<100	1132 (31.4%)	
100-200	828 (23.0%)	
200-1500	1321 (36.6%)	
>=1500	323 (9.0%)	
**Revised cardiac risk index**		
Low (≤1 point)	1476 (41.0%)	429 (41.8%)
Moderate (2 points)	1344 (37.3%)	396 (38.6%)
High (≥3 points)	783 (21.7%)	201 (19.6%)
**NSQIP MICA risk (%)**		
mean (sd)	1.84 (1.77)	1.90 (2.10)
median [iqr]	1.34 [0.58, 2.46]	1.24 [0.54, 2.61]
min to max	0.02 to 15.06	0.03 to 22.79
**Self-reported functional capacity**		
Stair climbing <1 floor	402 (11.2%)	198 (19.3%)
Inactive or regular physical activity ≤20 min/week activity	2218 (61.6%)	725 (70.7%)

©2023 Buse GL et al. *JAMA Network Open.*

eTable 2. Adjusted Odds ratios (OR) for in-hospital and 30-day MACE for RCRI-based models including NTproBNP or each of the assessed self-reported functional capacity measures

The 3 last columns present the results for models including both NTproBNP AND each one of the assessed self-reported functional capacity measures.

**In-hospital MACE**							
	**OR (95%CI)**	**OR (95%CI)**	**OR (95%CI)**	**OR (95%CI)**	**OR (95%CI)**	**OR (95%CI)**	**OR (95%CI)**
**RCRI**							
Low (≤1 point)	Reference	Reference	Reference	Reference	Reference	Reference	Reference
Moderate (2 points)	1.53 (0.88-2.66)	1.69 (0.98-2.93)	1.71 (0.99-2.97)	1.73 (1.00-3.00)	1.52 (0.88-2.64)	1.54 (0.89-2.68)	1.57 (0.90-2.73)
High (≥3 points)	1.91 (1.06-3.43)	2.49 (1.42-4.39)	2.57 (1.46-4.52)	2.41 (1.36-4.24)	1.88 (1.04-3.38)	1.92 (1.07-3.45)	1.85 (1.03-3.33)
**Age(years)**							
<65	Reference	Reference	Reference	Reference	Reference	Reference	Reference
65-74	1.15 (0.47-2.81)	1.17 (0.48-2.84)	1.18 (0.48-2.86)	1.25 (0.51-3.04)	1.17 (0.48-2.87)	1.17 (0.48-2.87)	1.22 (0.50-3.00)
>75	1.73 (0.73-4.13)	2.09 (0.89-4.95)	2.14 (0.90-5.04)	2.24 (0.95-5.29)	1.71 (0.72-4.07)	1.74 (0.73-4.14)	1.79 (0.75-4.29)
**NT-proBNP (pg/ml)**							
<100	Reference				Reference	Reference	Reference
100-200	0.96 (0.44-2.11)				0.97 (0.44-2.12)	0.96 (0.44-2.10)	0.94 (0.43-2.06)
200-1500	1.85 (0.99-3.44)				1.84 (0.99-3.44)	1.83 (0.98-3.41)	1.75 (0.94-3.27)
>1500	3.71 (1.81-7.61)				3.46 (1.68-7.15)	3.55 (1.71-7.37)	3.24 (1.57-6.69)
**METs**							
>=4		Reference			Reference		
<4		1.86 (1.10-3.16)			1.64 (0.95-2.80)		
**Stair climbing**							
<1 floor			Reference			Reference	
≥ 1 floor			0.65 (0.36-1.17)			0.80 (0.44-1.45)	
**Self-reported physical activity**							
Over 20 min/week				Reference			Reference
Inactive or low activity				2.40 (1.41-4.08)			2.15 (1.26-3.68)
**30-day MACE**							
	**OR (95%CI)**	**OR (95%CI)**	**OR (95%CI)**	**OR (95%CI)**	**OR (95%CI)**	**OR (95%CI)**	**OR (95%CI)**
**RCRI**							

Low (≤1 point)	Reference	Reference	Reference	Reference	Reference	Reference	Reference
Moderate (2 points)	1.29 (0.78-2.13)	1.44 (0.87-2.36)	1.46 (0.89-2.40)	1.46 (0.89-2.41)	1.28 (0.78-2.12)	1.31 (0.79-2.16)	1.31 (0.80-2.17)
High (≥3 points)	1.69 (1.00-2.86)	2.27 (1.37-3.77)	2.33 (1.41-3.86)	2.23 (1.34-3.71)	1.66 (0.98-2.82)	1.70 (1.00-2.88)	1.66 (0.98-2.81)
**Age(years)**							
<65	Reference	Reference	Reference	Reference	Reference	Reference	Reference
65-74	1.12 (0.49-2.58)	1.14 (0.50-2.60)	1.16 (0.51-2.65)	1.18 (0.52-2.71)	1.14 (0.50-2.62)	1.16 (0.50-2.66)	1.16 (0.51-2.67)
>75	1.75 (0.78-3.92)	2.18 (0.98-4.84)	2.20 (0.99-4.88)	2.29 (1.03-5.09)	1.73 (0.77-3.87)	1.76 (0.79-3.94)	1.79 (0.80-4.00)
**NT-proBNP (pg/ml)**							
<100	Reference				Reference	Reference	Reference
100-200	1.40 (0.69-2.86)				1.40 (0.69-2.86)	1.39 (0.68-2.83)	1.39 (0.68-2.83)
200-1500	2.18 (1.19-4.00)				2.18 (1.19-4.00)	2.14 (1.16-3.93)	2.11 (1.15-3.88)
>1500	4.71 (2.37-9.37)				4.44 (2.22-8.88)	4.38 (2.18-8.81)	4.33 (2.16-8.67)
**METs**							
>=4		Reference			Reference		
<4		1.76 (1.08-2.88)			1.54 (0.93-2.54)		
**Stair climbing**							
<1 floor			Reference			Reference	
≥ 1 floor			0.56 (0.34-0.94)			0.69 (0.41-1.17)	
**Self-reported physical activity**							
Over 20 min/week				Reference			Reference
Inactive or low activity				1.77 (1.12-2.80)			1.56 (0.98-2.48)

All models include a random intercept by country. *6/3597 patients had incomplete answers to the METs questions; the analyses on METs based on 86 in-hospital MACE in 3591 patients and 103 MACE in 3587 patients. **Regular physical activity to brisk walking, jogging or running, cycling, swimming, or vigorous sports at a comfortable pace or other activities requiring similar levels of exertion. CI: confidence interval; MACE: major adverse cardiac events; METs: metabolic equivalents; NTproBNP: N-terminal pro-Brain Natriuretic Peptide; OR: odds ratio RCRI: Revised Cardiac Risk Index.

eTable 3. Brier Score and ROC AUC for in-hospital and 30-day MACE from mixed effect logistic regression models based the addition of NTproBNP to models including RCRI+age+functional capacity measures

	Inhospital MACE			30-day MACE		
Models combining self-reported functional capacity and NTproBNP	Brier Score	ROC AUC (9.5%CI)	p-value to corres-ponding model without NTproBNP	Brier Score	ROC AUC (95%CI)	p-value to corres-ponding model without NTproBNP
RCRI+age+*<4METs** **+NTproBNP**	0.023	0.741 (0.688-0.795)	0.03	0.027	0.724 (0.674-0.774)	0.02
RCRI+age+< *1 floor stairs* **+NTproBNP**	0.023	0.739 (0.686-0.792)	0.18	0.027	0.723 (0.673-0.773)	0.03
RCRI+age+*<20 min/w regular physical activity*****+NTproBNP**	0.023	0.750 (0.699-0.801)	0.04	0.027	0.726 (0.677-0.774)	0.03

All models include a random intercept by country. *6/3597 patients had incomplete answers to the METs questions; the analyses on METs based on 86 in-hospital MACE in 3591 patients and 103 MACE in 3587 patients. **Regular physical activity to brisk walking, jogging or running, cycling, swimming, or vigorous sports at a comfortable pace or other activities requiring similar levels of exertion.

AUC: area under the curve; CI: confidence interval; MACE: major adverse cardiac events; METs: metabolic equivalents; NTproBNP: N-terminal pro Brain Natriuretic Peptide; RCRI: Revised Cardiac Risk Index;

eTable 4. Weighted comparison and benefit equivalent for MACE for the addition of NTproBNP to models including RCRI+age+functional capacity measures

In-hospital MACE incidence is 2.4%, 30-day MACE incidence is 2.9% misclassification cost is set at 10%.

	**In-hospital MACE**		**30-day MACE**	
**Comparison between**	**Weighted comparison**	**Benefit equivalent (per 1000 patients)***	**Weighted comparison**	**Benefit equivalent (per 1000 patients)***
RCRI+age+4MET+ **NTproBNP** vs RCRI+age+4METs*	0.046	1	0.148	4
RCRI+age+< 1 floor of stairs+ **NTproBNP** vs RCRI+age+< 1 floor of stairs	0.268	6	0.149	4
RCRI+age+physical activity+**NTproBNP** vs RCRI+age+physical activity	0.361	9	0.334	10

eTable 5. Adjusted Odds ratios (OR) for in-hospital and 30-day MACE for NSQIP MICA-based models including NTproBNP or each of the assessed self-reported functional capacity measures

The 3 last columns present the results for models including both NTproBNP AND each one of the assessed self-reported functional capacity measures.

In-hospital MACE							
	**OR (95%CI)**	**OR (95%CI)**	**OR (95%CI)**	**OR (95%CI)**	**OR (95%CI)**	**OR (95%CI)**	**OR (95%CI)**
**NSQIP MICA risk (%)**	1.05 (0.95-1.17)	1.13 (1.02-1.24)	1.13 (1.02-1.25)	1.12 (1.01-1.24)	1.06 (0.95-1.18)	1.06 (0.95-1.18)	1.05 (0.94-1.18)
**NT-proBNP (pg/ml)**							
<100	Reference				Reference	Reference	Reference
100-200	1.06 (0.49-2.32)				1.06 (0.48-2.31)	1.05 (0.48-2.30)	1.03 (0.47-2.25)
200-1500	2.21 (1.21-4.06)				2.17 (1.18-4.00)	2.17 (1.18-3.99)	2.07 (1.13-3.81)
>1500	4.95 (2.48-9.86)				4.51 (2.25-9.04)	4.69 (2.32-9.46)	4.25 (2.12-8.52)
**METs**							
>=4		Reference			Reference		
<4		2.07 (1.23-3.50)			1.73 (1.01-2.95)		
**Stair climbing**							
<1 floor			Reference			Reference	
≥ 1 floor			0.59 (0.33-1.05)			0.77 (0.43-1.40)	
**Self-reported physical activity**							
Over 20 min/week				Reference			Reference
Inactive or low activity				2.51 (1.48-4.25)			2.17 (1.27-3.70)
**30-day MACE**							
	**OR (95%CI)**	**OR (95%CI)**	**OR (95%CI)**	**OR (95%CI)**	**OR (95%CI)**	**OR (95%CI)**	**OR (95%CI)**
**NSQIP MICA risk (%)**	1.06 (0.97-1.17)	1.14 (1.04-1.24)	1.14 (1.04-1.24)	1.14 (1.04-1.24)	1.06 (0.97-1.17)	1.07 (0.97-1.17)	1.06 (0.96-1.17)
**NT-proBNP (pg/ml)**							
<100	Reference				Reference	Reference	Reference
100-200	1.54 (0.76-3.13)				1.53 (0.75-3.12)	1.52 (0.75-3.09)	1.52 (0.74-3.08)
200-1500	2.58 (1.42-4.67)				2.54 (1.40-4.60)	2.50 (1.38-4.54)	2.48 (1.37-4.49)
>1500	6.11 (3.15-11.84)				5.64 (2.89-10.98)	5.59 (2.85-10.97)	5.55 (2.85-10.82)
**METs**							
>=4		Reference			Reference		

©2023 Buse GL et al. *JAMA Network Open.*

<4		1.96 (1.21-3.19)			1.63 (0.99-2.68)		
**Stair climbing**							
<1 floor			Reference			Reference	
≥ 1 floor			0.50 (0.30-0.84)			0.67 (0.39-1.12)	
**Self-reported physical activity**							
Over 20 min/week				Reference			Reference
Inactive or low activity				1.87 (1.18-2.94)			1.59 (1.00-2.52)

All models include a random intercept by country. *6/3597 patients had incomplete answers to the METs questions; the analyses on METs based on 86 in-hospital MACE in 3591 patients and 103 MACE in 3587 patients. **Regular physical activity to brisk walking, jogging or running, cycling, swimming, or vigorous sports at a comfortable pace or other activities requiring similar levels of exertion. CI: confidence interval; MACE: major adverse cardiac events; METs: metabolic equivalents; NTproBNP: N-terminal pro-Brain Natriuretic Peptide; NSQIP MICA: National Surgical Quality Improvement Program, Risk calculator for Myocardial Infarction and Cardiac Arrest; OR: odds ratio.

eTable 6. Brier Score and ROC AUC for MACE from mixed effect logistic regression models based on for the addition of NTproBNP to models including NSQIP MICA+functional capacity measures

	Inhospital MACE			30-day MACE		
Models combining self-reported functional capacity and NTproBNP	Brier Score	ROC AUC (95%CI)	p-value to corres-ponding model without NTproBNP	Brier Score	ROC AUC (95%CI)	p-value to corres-ponding model without NTproBNP
NSQIP MICA+<4METs* **+NTproBNP**	0.023	0.737 (0.686-0.787)	0.05~~3~~	0.027	0.724 (0.675-0.773)	0.02~~4~~
NSQIP MICA+< 1 floor stairs **+NTproBNP**	0.023	0.734 (0.684-0.784)	0.04~~2~~	0.027	0.720 (0.670-0.769)	0.04~~0~~
NSQIP MICA+<20 min/w regular physical activity**+NTproBNP**	0.023	0.742 (0.691-0.793)	0.05~~48~~	0.027	0.726 (0.678-0.774)	0.03~~26~~

©2023 Buse GL et al. *JAMA Network Open.*

eTable 7. Weighted comparison and benefit equivalent for in-hospital and for 30-day MACE for NSQIP-MICA-based models

In-hospital MACE incidence is 2.4%, 30-day MACE incidence is 2.9%, and misclassification cost is set at 10%.

	**In-hospital MACE**		**30-day MACE**	
Comparison between	Weighted comparison	Benefit equivalent (per 1000 patients)*	Weighted comparison	Benefit equivalent (per 1000 patients)*
NSQIP-MICA+**NTproBNP** vs NSQIP-MICA+**4MET***	-0.003	0	0.150	4
NSQIP-MICA+**NTproBNP** vs NSQIP-MICA+**< 1 floor of stairs**	-0.004	0	0.149	4
NSQIP-MICA+**NTproBNP** vs NSQIP-MICA+**physical activity**	-0.003	0	0.148	4
**Models combining self-reported functional capacity and NTproBNP**				
NSQIP-MICA+4MET**+NTproBNP** vs NSQIP-MICA+4MET	0.18	4	0.149	4
NSQIP-MICA+< 1 floor of stairs **+NTproBNP** vs NSQIP-MICA+< 1 floor of stairs	0.04	1	0.148	4

©2023 Buse GL et al. *JAMA Network Open.*

NSQIP-MICA+physical activity+**NTproBNP** vs NSQIP-MICA+physical activity	0.176	4	0.299	9

eFigure 1. Decision curves of various RCRI-based models for in-hospital MACE

Green: a,b)RCRI+age+**NTproBNP**;

Blue: a) RCRI+age+**4 METs**; b) RCRI+age+**stair climbing <1 floor**

Pink: a,b) RCRI+age+**regular physical activity**

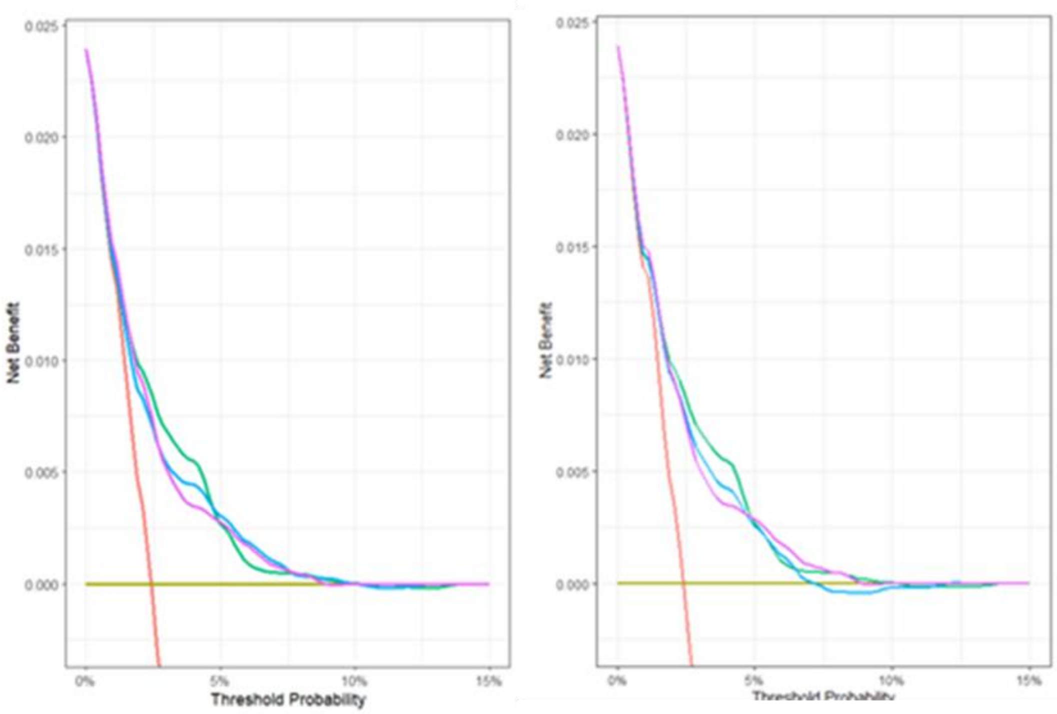

©2023 Buse GL et al. *JAMA Network Open.*

eFigure 2. Decision curves of various RCRI-based models combining NTproBNP and self-reported functional capacity measures for in-hospital MACE

Green: a) RCRI+age+*4 METs*+**NTproBNP**; b) RCRI+age+ *<1 floor*+**NTproBNP**; c) RCRI+age+*regular physical activity*+**NTproBNP**

Pink: a) RCRI+age+**4 METs**; b) RCRI+age+ **<1 floor**; c) RCRI+age+**regular physical activity**

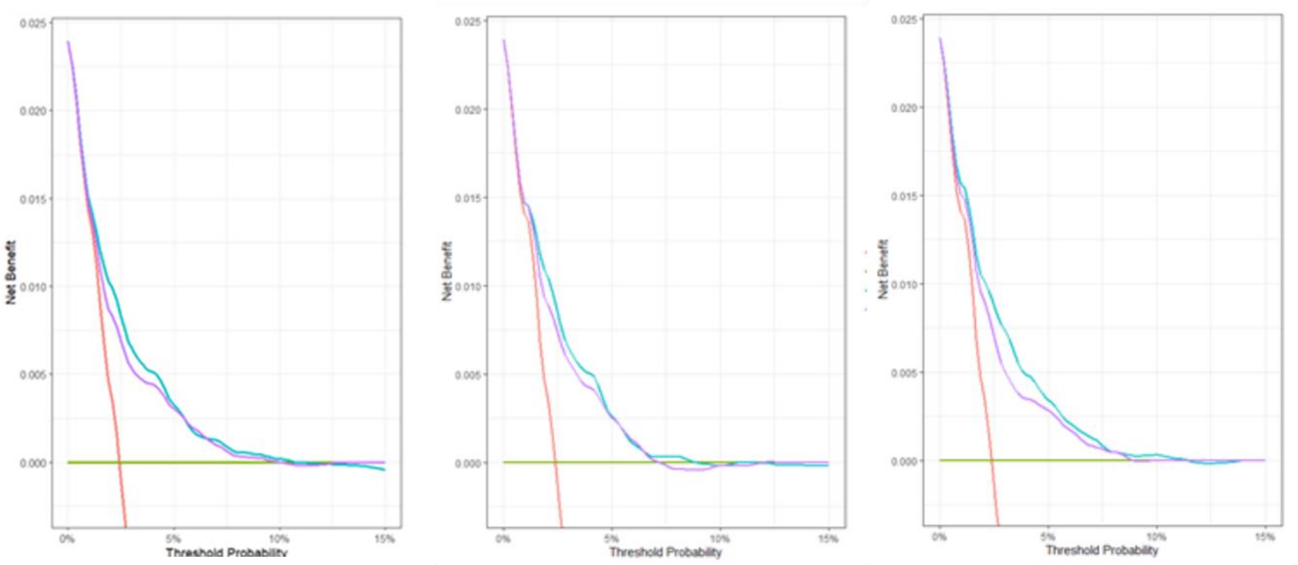

©2023 Buse GL et al. *JAMA Network Open.*

eFigure 3. Decision curves of various NSQIP MICA-based models for in-hospital MACE

Green: a,b) NSQIP MICA+**NTproBNP**;

Blue: a) NSQIP MICA +**4 METs**; b) NSQIP MICA +**stair climbing <1 floor**

Pink: a,b) NSQIP MICA +**regular physical activity**

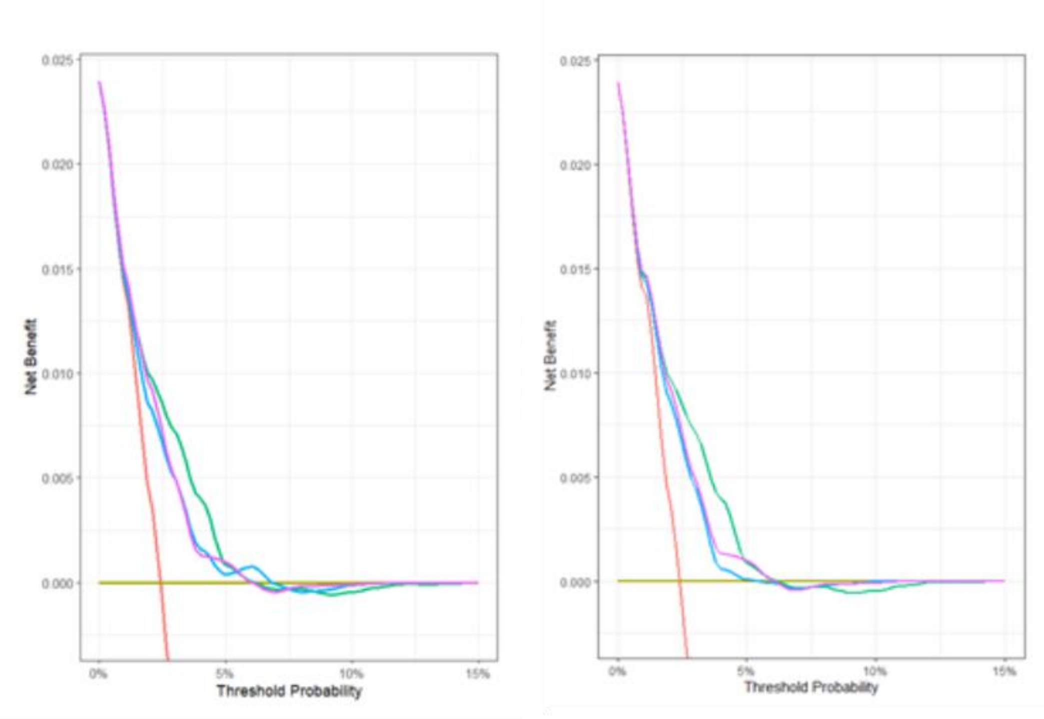

©2023 Buse GL et al. *JAMA Network Open.*

eFigure 4. Decision curves of various NSQIP MICA-based models combining NTproBNP and self-reported functional capacity measures for in-hospital MACE

Green: a) NSQIP MICA +*4 METs*+**NTproBNP**; b) NSQIP MICA + *<1 floor*+**NTproBNP**; c) NSQIP MICA +*regular physical activity*+**NTproBNP**

Pink: a) NSQIP MICA **+4 METs**; b) NSQIP MICA + **<1 floor**; c) NSQIP MICA +**regular physical activity**

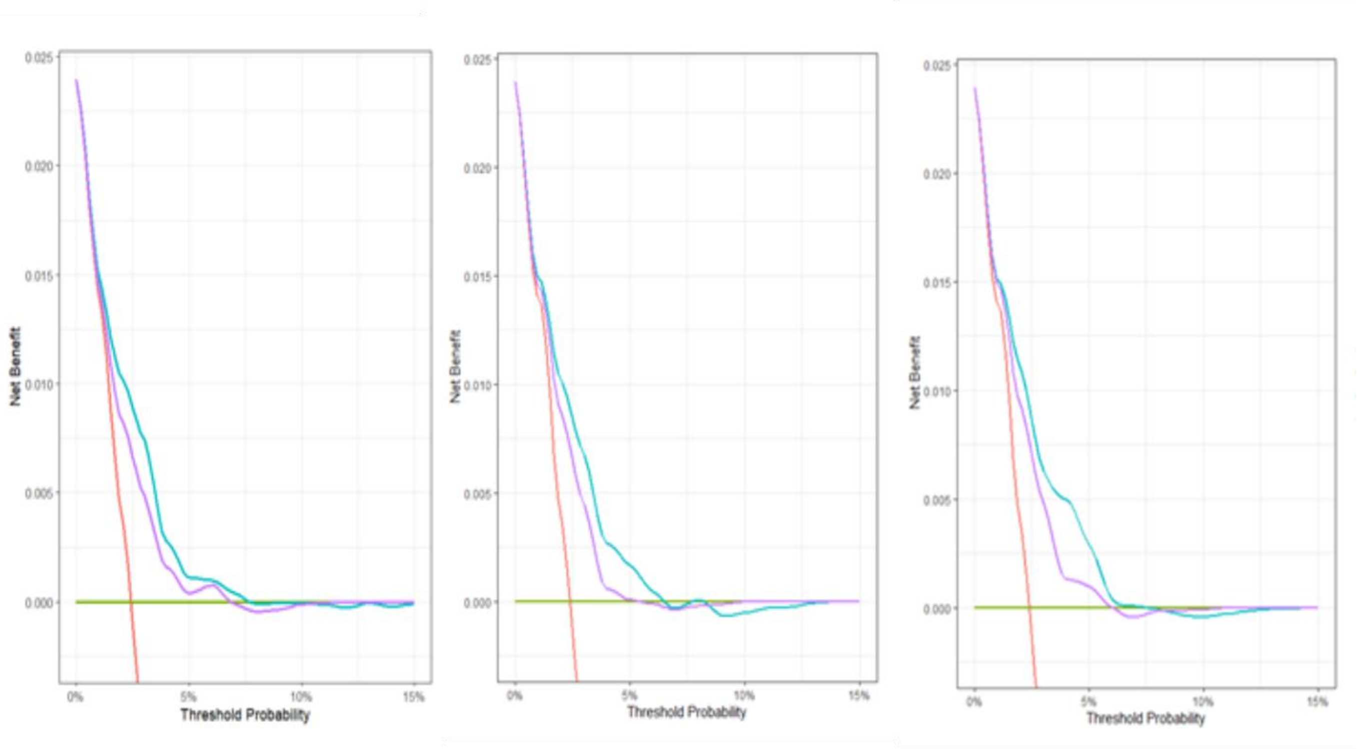

©2023 Buse GL et al. *JAMA Network Open.*
