# Supplementary material for: NT-proBNP or Self-Reported Functional Capacity in Estimating Risk of Cardiovascular Events After Noncardiac Surgery
Source: JAMA Netw Open. 2023 Nov 8;6(11):e2342527. doi: 10.1001/jamanetworkopen.2023.42527 (PMC10632953; doi:10.1001/jamanetworkopen.2023.42527)
Supplement: Supplement 2. — Nonauthor Collaborators [file jamanetwopen-e2342527-s002.pdf]

Supplemental Online Content: Nonauthor Collaborators
*First name, last name, and suffix (if applicable) are required and will appear in PubMed.

*Group Name(s): METREPAIR NTproBNP subcohort Investigators

*First Name and Middle Initial(s)	*Last Name	*Suffix (eg, Jr, III)	Academic Degrees	Institution	Location (city, state/province, country)	Role or Contribution, eg, chair, principal investigator	Group (if more than 1 Group listed in the byline) and/or Subgroup (eg, Steering Committee)
Johann	Kemper		MD	University Hospital Düsseldorf, Heinrich Heine University	Düsseldorf, Germany	local investigator	METREPAIR Investigator
Lena	Kohaupt		MD	University Hospital Düsseldorf, Heinrich Heine University	Düsseldorf, Germany	local investigator	METREPAIR Investigator
Jette	Witzler			University Hospital Düsseldorf, Heinrich Heine University	Düsseldorf, Germany	local investigator	METREPAIR Investigator
Michael	Tuzhikov		MD	University Hospital Düsseldorf, Heinrich Heine University	Düsseldorf, Germany	local investigator	METREPAIR Investigator
Sebastian	Roth		MD	University Hospital Düsseldorf, Heinrich Heine University	Düsseldorf, Germany	local investigator	METREPAIR Investigator
Alexandra	Stroda		MD	University Hospital Düsseldorf, Heinrich Heine University	Düsseldorf, Germany	local investigator	METREPAIR Investigator
Rene	MPembele		MD	University Hospital Düsseldorf, Heinrich Heine University	Düsseldorf, Germany	local investigator	METREPAIR Investigator
Cornelia	Schultze		MD	Hannover Medical School	Hannover, Germany	local investigator	METREPAIR Investigator
Nele	Verbarg		MD	Hannover Medical School	Hannover, Germany	local investigator	METREPAIR Investigator
Christian	Gehrke		MD	Hannover Medical School	Hannover, Germany	local investigator	METREPAIR Investigator
Florian	Espeter		MD	Heidelberg University Hospital	Heidelberg, Germany	local investigator	METREPAIR Investigator
Benedikt	Russe		MD	Heidelberg University Hospital	Heidelberg, Germany	local investigator	METREPAIR Investigator
Markus A.	Weigand		Professor	Heidelberg University Hospital	Heidelberg, Germany	local investigator	METREPAIR Investigator
Raphael	Pirzer		MD	Universitätsklinikum Augsburg	Augsburg, Germany	local investigator	METREPAIR Investigator
Patric Rene	Rach		MD	Universitätsklinikum Augsburg	Augsburg, Germany	local investigator	METREPAIR Investigator
Claudia	Neumann		MD	University Hospital Bonn	Bonn, Germany	local investigator	METREPAIR Investigator
Christoph	Sponhol		MD	Jena University Hospital	Jena, Germany	local investigator	METREPAIR Investigator
Melissa	Carollo		MD	ASST Settelaghi – Ospedale di Circolo e Fondazione Macchi	Varese, Italy	local investigator	METREPAIR Investigator

Supplemental Online Content: Nonauthor Collaborators
*First name, last name, and suffix (if applicable) are required and will appear in PubMed.

***First Name and Middle Initial(s)**	***Last Name**	***Suffix (eg, Jr, III)**	Academic Degrees	Institution	Location (city, state/province, country)	Role or Contribution, eg, chair, principal investigator	Group (if more than 1 Group listed in the byline) and/or Subgroup (eg, Steering Committee)
Fiorenza	Toso		MD	ASST Settelaghi – Ospedale di Circolo e Fondazione Macchi	Varese, Italy	local investigator	METREPAIR Investigator
Alessandro	Bacuzzi		MD	ASST Settelaghi – Ospedale di Circolo e Fondazione Macchi	Varese, Italy	local investigator	METREPAIR Investigator
Marta G.	Servén		MD	Hospital de la Santa Creu i Sant	Barcelona, Spain	local investigator	METREPAIR Investigator
Anna	Artigas Soler		MD	Corporacio sanitaria Parc Tauli	Sabadell, Spain	local investigator	METREPAIR Investigator
Morena	Basso		MD	Corporacio sanitaria Parc Tauli	Sabadell, Spain	local investigator	METREPAIR Investigator
Anna	Peig Font		MD	Corporacio sanitaria Parc Tauli	Sabadell, Spain	local investigator	METREPAIR Investigator
Jara	Torrente-Perez		MD	Hospital Universitario La Princesa	Madrid, Spain	local investigator	METREPAIR Investigator
Maria Isabel	Fores		MD	Hospital Arnau De Vilanova–Lliria	Valencia, Spain	local investigator	METREPAIR Investigator
Serban	Bubenek-Turconi		MD	Emergency Institute for CardioVascular Diseases Prof. Dr. C.C.Iliescu	Bucharest, Romania	local investigator	METREPAIR Investigator
Liana	Vale		MD	Emergency Institute for CardioVascular Diseases Prof. Dr. C.C.Iliescu	Bucharest, Romania	local investigator	METREPAIR Investigator
Patrick	Wanner		MD	Kantonsspital St Gallen	St Gallen, Switzerland	local investigator	METREPAIR Investigator
Mirjana	Djurdjevic		RN	Kantonsspital St Gallen	St Gallen, Switzerland	local investigator	METREPAIR Investigator
Sandra	Nuth		MD	Kantonsspital St Gallen	St Gallen, Switzerland	local investigator	METREPAIR Investigator
Esther	Seeberger		RN	Basel University Hospital	Basel, Switzerland	local investigator	METREPAIR Investigator
Firmin	Kamber		MD	Basel University Hospital	Basel, Switzerland	local investigator	METREPAIR Investigator
Thomas Jan	Gerber		MD	Kantonsspital Winterthur	Winterthur, Switzerlanc	local investigator	METREPAIR Investigator
Daniela	Schneebeli		MD	Kantonsspital Winterthur	Winterthur, Switzerlanc	local investigator	METREPAIR Investigator
Sina	Grape		MD	Hôpital du Valais	Sion, Switzerland	local investigator	METREPAIR Investigator
Bernardo	Bollen Pinto		MD PhD	Geneva University Hospitals	Geneva, Switzerland	local investigator	METREPAIR Investigator
Igor	Karolak		MD	Pomeranian Medical University	Szczecin, Poland	local investigator	METREPAIR Investigator
Kacper	Lechowicz		MD	Pomeranian Medical University	Szczecin, Poland	local investigator	METREPAIR Investigator
Sylwester	Drozdzal		MD	Pomeranian Medical University	Szczecin, Poland	local investigator	METREPAIR Investigator

Supplemental Online Content: Nonauthor Collaborators
*First name, last name, and suffix (if applicable) are required and will appear in PubMed.

***First Name and Middle Initial(s)**	***Last Name**	***Suffix (eg, Jr, III)**	Academic Degrees	Institution	Location (city, state/province, country)	Role or Contribution, eg, chair, principal investigator	Group (if more than 1 Group listed in the byline) and/or Subgroup (eg, Steering Committee)
Anna	Kluzik		MD	Heliodor Swiecicki Clinical Hospital at Poznan University of Medical Sciences	Poznan, Poland	local investigator	METREPAIR Investigator
Felix	van Lier		MD	Erasmus Medical Centre	Rotterdam, Netherlands	local investigator	METREPAIR Investigator
Andre	Carrao		MD	Hospital Beatriz Ângelo	Lisbon, Portugal	local investigator	METREPAIR Investigator
Daniela	Ribeiro		MD	Hospital Beatriz Ângelo	Lisbon, Portugal	local investigator	METREPAIR Investigator
Nuno	Santos		MD	Hospital de Santo Espírito da Ilha Terceira, E.P.E.R.	Angra de Heroismo, Portugal	local investigator	METREPAIR Investigator
Andrew	Drummond		MD	The Royal Oldham Hospital - The Pennine Acute Hospitals NHS Trust	Oldham, UK	local investigator	METREPAIR Investigator
Vladislav	Belskii		MD	Privolzhskiy District Medical Center	Nizhniy Novgorod, Russia	local investigator	METREPAIR Investigator
