# Supplementary material for: NT-proBNP or Self-Reported Functional Capacity in Estimating Risk of Cardiovascular Events After Noncardiac Surgery
Source: JAMA Netw Open. 2023 Nov 8;6(11):e2342527. doi: 10.1001/jamanetworkopen.2023.42527 (PMC10632953; doi:10.1001/jamanetworkopen.2023.42527)
Supplement: Supplement 3. — Data Sharing Statement [file jamanetwopen-e2342527-s003.pdf]

# Data Sharing Statement

Lurati Buse. NTproBNP or Self-Reported Functional Capacity in Projecting Cardiovascular Events After Noncardiac Surgery. *JAMA Netw Open*. Published November 08, 2023. doi:10.1001/jamanetworkopen.2023.42527

**Data**
**Data available:** Yes
**Data types:** Deidentified participant data
**How to access data:** giovanna.luratibuse@med.uni-duesseldorf.de; research@esaic.org; data will be shared upon reasonable request and in line with the rules stated in the study protocol
**When available:** With publication

**Supporting Documents**
**Document types:** Informed consent form
**How to access documents:** esaic.org
**When available:** With publication

**Additional Information**
**Who can access the data:** researchers whose proposed use of the data has been approved
**Types of analyses:** for a specified purpose after approval of the steering committee
**Mechanisms of data availability:** after approval of a proposal and with a signed data access agreement
